# Applying the Spatial Transmission Network to the Forecast of Infectious Diseases Across Multiple Regions

**DOI:** 10.3389/fpubh.2022.774984

**Published:** 2022-03-11

**Authors:** Huimin Wang, Jianqing Qiu, Cheng Li, Hongli Wan, Changhong Yang, Tao Zhang

**Affiliations:** ^1^Department of Epidemiology and Health Statistics, West China School of Public Health and West China Fourth Hospital, Sichuan University, Chengdu, China; ^2^Sichuan Center for Disease Control and Prevention, Chengdu, China

**Keywords:** spatial transmission network, infectious disease, vector autoregressive moving average, SEIRS, long-short term memory

## Abstract

**Objective:**

Timely and accurate forecast of infectious diseases is essential for achieving precise prevention and control. A good forecasting method of infectious diseases should have the advantages of *interpretability, feasibility*, and *forecasting performance*. Since previous research had illustrated that the spatial transmission network (STN) showed good interpretability and feasibility, this study further explored its forecasting performance for infectious diseases across multiple regions. Meanwhile, this study also showed whether the STN could overcome the challenges of model rationality and practical needs.

**Methods:**

The construction of the STN framework involved three major steps: the spatial kluster analysis by tree edge removal (SKATER) algorithm, structure learning by dynamic Bayesian network (DBN), and parameter learning by the vector autoregressive moving average (VARMA) model. Then, we evaluated the forecasting performance of STN by comparing its accuracy with that of the mechanism models like susceptible-exposed-infectious-recovered-susceptible (SEIRS) and machine-learning algorithm like long-short-term memory (LSTM). At the same time, we assessed the robustness of forecasting performance of STN in high and low incidence seasons. The influenza-like illness (ILI) data in the Sichuan Province of China from 2010 to 2017 were used as an example for illustration.

**Results:**

The STN model revealed that ILI was likely to spread among multiple cities in Sichuan during the study period. During the whole study period, the forecasting accuracy of the STN (mean absolute percentage error [MAPE] = 31.134) was significantly better than that of the LSTM (MAPE = 41.657) and the SEIRS (MAPE = 62.039). In addition, the forecasting performance of STN was also superior to those of the other two methods in either the high incidence season (MAPE = 24.742) or the low incidence season (MAPE = 26.209), and the superiority was more obvious in the high incidence season.

**Conclusion:**

This study applied the STN to the forecast of infectious diseases across multiple regions. The results illustrated that the STN not only had good accuracy in forecasting performance but also indicated the spreading directions of infectious diseases among multiple regions to a certain extent. Therefore, the STN is a promising candidate to improve the surveillance work.

## Introduction

The outbreak of infectious diseases poses a serious threat to public health and imposes a heavy burden on the global economy. According to recent research studies, there were around 291,000–646,000 deaths caused by seasonal influenza-related respiratory illnesses worldwide every year (1, 2). The US Centers for Disease Control and Prevention (CDC) estimated that from October 1, 2019 to April 4, 2020, there were 39,000,000–56,000,000 flu infections and 24,000–62,000 flu deaths in the US (3). In addition, the WHO assessed that the first influenza pandemic in this century damaged the global economy by around $800 billion (4).

In view of the severe threats of infectious diseases, consistently improving the infectious disease surveillance work is the top priority for health promotion (5). How to utilize the surveillance data to achieve accurate and reliable forecasting in the early stage of infectious diseases is an important basis for formulating corresponding prevention and control strategies, so it has attracted the attention of a large number of researchers. Specifically, good forecasting for infectious diseases requires three main properties: (1) *interpretability*, that is, the model reflects the epidemic features of infectious diseases in order to guarantee the forecasted results to make sense in practice, (2) *feasibility*, which involves the support of statistical software (such as SPSS, SAS, and R), the availability of data, and the needs from disease surveillance, etc., and (3) *forecasting performance*, the inherent ability of the model to accurately forecast the future epidemics of infectious diseases, so as to help propose precise prevention and control measures and to allocate limited resources in the most needed places. To this end, previous research proposed the spatial transmission network (STN) and showed its good interpretability and feasibility for the transmission mechanism of diseases (6). However, the last but most important work of forecasting performance assessment was left to be done. Therefore, the purpose of this study was to further explore the forecasting performance of the STN for infectious diseases across multiple regions.

However, there were two major challenges when exploring the forecasting performance of STN. The first challenge was related to the rationality of the model. The STN model essentially involved spatial clustering analysis, structure learning, and parameter learning, where the vector autoregressive moving average (VARMA) model was adopted for parameter learning. Although it seemed natural to apply the VARMA model to forecasting, the rationality remained quite unclear in the spatio-temporal settings, especially when compared with mechanism models (7, 8) and machine learning methods (9). For example, as classic representatives of mechanism models, the compartmental models, such as susceptible-infectious-recovered-susceptible (SIRS) (10, 11), susceptible-exposed-infectious-recovered (SEIR) (12), and susceptible-exposed-infectious-recovered-susceptible (SEIRS) (13), have been widely applied in the field of influenza prediction. Meanwhile, the machine learning methods are also playing more and more important roles in infectious disease forecasting. Svitlana et al. established and evaluated the capability of long-short term memory (LSTM) for nowcasting (predicting in “real time”) and forecasting (predicting the future) of influenza-like illness (ILI) dynamics, which found that the neural network structure that relied on LSTM units outperformed the traditional regression model (14). Therefore, the rationality of the VARMA model in infectious disease forecasting should be qualified by comparing its performance with those of the machine learning models and mechanism models.

In addition, the second challenge came from practical needs. To be specific, both spatial and temporal transmission of infectious diseases are influenced by many external factors, such as the effective distance between the cities, population size, and transportation. Previous research showed fantastic applications on how such factors could be used to improve forecasting performance (15). In addition, another inspiring study suggested that, in order to verify the robustness of the model when dealing with seasonal infectious diseases, its forecasting performance should be verified in both high and low incidence seasons (16). All these contributions shed light upon a systematic way to build the forecasting framework based on the STN model.

This study will show whether the STN could overcome the above two challenges and contribute to the forecast of infectious diseases across multiple regions. The sentinel surveillance data of ILI in the Sichuan Province of China from 2010 to 2017 was used as an example for illustration. Sichuan is one of the core regions for influenza surveillance in China. The representativeness of these areas not only guaranteed the applicability of this method in these regions but also offered evidence for future application to other regions.

## Materials and Methods

### Data Collection and Preparation

Ever since the outbreak of influenza A (H1N1) in 2009, China has established a nationwide ILI sentinel surveillance system, which consists of 193 sentinel hospitals in 30 provinces at present. The identification of patients with ILI is based on the standard case definition of the WHO, that is, body temperature ≥38°C with either cough or sore throat, in the absence of an alternative diagnosis (17). Besides, the ILI% was widely used in influenza surveillance to reflect the local intensity of the influenza epidemic, where ILI% = (the number of ILI cases in outpatient and emergency departments)/(the total number of outpatient and emergency cases) ×100% (18, 19). Taking *n* places as an example, *x*_1_(*t*), *x*_2_(*t*), ⋯ , *x*_*n*_(*t*) denoted the ILI% values of the place *n* at the *t*-th week. Accordingly, all the observed data at the *t*-th week could be noted as *x*_*t*_ = {*x*_1_(*t*), *x*_2_(*t*), ⋯ , *x*_*n*_(*t*)}, where *x*_*t*_ is a vector with *n* series components (boldface notation indicated vectors and matrices hereafter). As for this study, the weekly ILI% time series data came from each city of Sichuan Province from 2010 to 2017, so *t* ranged from 1 to 416. Such sample size was large enough relative to the complexity of the models utilized below, since the maximum degrees of freedom of the STN, LSTM, and SEIRS model employed by this study were no more than 40. In addition, Zhang et al. reported that at least 3 years of weekly historical data were required for VARMA-type models to assure the quality of infectious diseases surveillance (20). Therefore, it was plausible that the sample size in this study could support the model estimation.

Furthermore, the application of STN required either each univariate time series to be *weak stationary* (i.e., the mean and covariance matrices of the time series are independent on time), or the multivariate time series to meet the *cointegration* condition (i.e., a linear combination of individual unit-root non-stationary time series becomes a stationary series), where the weak stationary and the cointegration conditions could be checked by the unit-root test and cointegration test, respectively (21). If both conditions were violated, one should seek other time series analysis techniques for the solution. For the sake of clarity, this study only focused on situations when both conditions were satisfied, and readers who might be interested in more complicated situations could refer to the works of other researchers (22).

### The Construction of STN

The construction of STN mainly consisted of three steps: spatial clustering analysis, structure learning, and parameter learning. First, due to the heterogeneity of ILI% data in Sichuan Province, the spatial kluster analysis by tree edge removal (SKATER) algorithm was used to divide the whole area into several homogeneous clusters. Second, the structural information of STN (i.e., the existence and direction of disease transmission) among cities in each cluster was identified from the original data through structure learning. Lastly, the parameters of STN that measured the intensity of disease transmission between different cities were estimated by parameter learning. After the construction of STN, the future incidence of infectious diseases could be forecasted.

#### Spatial Clustering Analysis

To avoid the curse of dimensionality in modeling and to make the results more interpretable, the SKATER algorithm was used for spatial clustering analysis. This is a graph clustering algorithm based on the minimum spanning tree, which obtains the clustering result that satisfies the spatial adjacency constraint by cutting off the edges of the minimum spanning tree (23). Specifically, after standardizing the ILI%, population density (people per-square kilometer), and the number of domestic tourists (10,000 person-times), the Euclidean distance between the two regions was calculated to obtain the difference matrix. The root adjacency method was used to constrain the adjacency matrix, the minimum clustering area size was set to two regions, and the result of cluster division was finally obtained.

#### The Structure Learning of STN

The first-order conditional dependencies approximation for dynamic Bayesian network (G1DBN) was used to build the structure of STN (24). It implemented the structural learning in a two-step way. Specifically, it first learned a directed acyclic graph from the first-order partial dependence relationships among the time series of different regions. Then, it optimized the graph from the first step to infer the network structure of the STN. As a result, the estimated STN structure would include a set of nodes to represent each city and pairs of edges to indicate which cities have an impact on others in influenza transmission (6).

#### The Parameter Learning of STN

We used the VARMA model for parameter learning, which was a flexible modeling framework that could comprehensively describe and predict the dynamic relations among each component of multivariate time series (25). Taking the *n*-dimensional as an example, a general VARMA (*p, q*) model can be written as:


(1) $$\begin{array}{r} x_{t}=a_{0}+\overset{p}{\underset{i=1}{\sum }}a_{i}x_{t-i}+\varepsilon_{t}-\overset{q}{\underset{j=1}{\sum }}b_{j}\varepsilon_{t-j}. \end{array}$$

Where, *p* and *q* are non-negative integers, ***a***_0_ is an *n*-dimensional constant vector, ***a***_*i*_ and ***b***_*j*_ are *n* × *n* constant matrices, and {**ε**_*t*_} is a sequence of independent and identically distributed random vectors. In addition, **ε**_*t*_ is the white noise sequence and the residual of the model fitting, and $\overset{q}{\underset{j=1}{\sum }}{\text{b}}_{j}\varepsilon_{t-j}$ is a moving average (MA) term.

The model-fitting step was comprised of order determination and parameter estimation. First, in the part of order selection, the values of *p* and *q* in Equation (1) were determined. Tiao and Tsay proposed to use the two-way *p*-value table for extended cross-correlation matrices to specify the order (*p, q*) (26). Once the orders were determined, the parameters of the VARMA model could be estimated by the conditional likelihood method. However, the VARMA model may encounter a problem of identifiability, which means that the coefficients may not be uniquely determined. To solve this problem, this study used the Kronecker index approach to perform structural specification of the VARMA model (25).

### The Forecast of STN

We applied the minimum mean-squared error criterion for the forecasts of the VARMA (*p, q*) time series *x*_*t*_. For VARMA (*p, q*) model, the *l*-step ahead forecast of *x*_*t*+*l*_ at the forecast origin *t* is:


(2) $$\begin{array}{r} x_{t}\left(l\right)=E\left(x_{t+l}|F_{t}\right)=a_{0}+\overset{p}{\underset{i=1}{\sum }}a_{i}{\text{X}}_{t}\left(l-i\right). \end{array}$$

A more convenient expression based on MA appears as:


(3) $$\begin{array}{r} x_{t}\left(l\right)=E\left(x_{t+l}|F_{t}\right)=\overset{\infty }{\underset{k=0}{\sum }}\psi_{l+k}\varepsilon_{t-k}. \end{array}$$

The prediction of the minimum mean-squared error of *x*_*t*+*l*_ is the conditional expectation of *x*_*t*+*l*_ at a given *F*_*t*_. We denote *F*_*t*_ and the information available at *t* and **ψ**_*l*_ as the coefficient matrices. The *l*-step ahead forecast error is Equation (4). Consequently, the covariance matrix of *l*-step ahead forecast error is Equation (5):


(4) $$\begin{array}{r} e_{t}\left(l\right)=\varepsilon_{t+l}+\overset{l-1}{\underset{k=1}{\sum }}\psi_{k}\varepsilon_{t+l-k}, \end{array}$$

(5) $$\begin{array}{r} Cov\left[e_{t}\left(l\right)\right]=\sum_{\varepsilon }+\overset{t-1}{\underset{k=1}{\sum }}\psi_{k}\sum_{\varepsilon }\psi_{k}^{T}, \end{array}$$

where Equation (4) shows that the forecast error tends to the random part of *x*_*t*_ as *l* increases. When *l* approaches 0, *x*_*t*_(*l*) tends to *E*(*x*_*t*_). Besides, Equation (5) shows that, as *l* approaches infinity, *Cov*[*e*_*t*_(*l*)] converges to *Cov*(*x*_*t*_). As the time lag increases, the dynamic dependence of the stationary reversible VARMA model decays exponentially to 0. Equation (5) also provides an effective method for calculating the covariance matrix of the forecast error.

### The Comparison Models

Since it has been already reported that the forecasting performance of the VARMA model was superior to that of the univariate time series model (27), this study chose another two mainstream methods, SEIRS and LSTM, as comparison models. The two methods respectively represented the mechanism models and machine learning methods, which could serve as benchmarks for forecasting performance comparison.

#### The SEIRS Model

The SEIRS model, as a type of mechanism model, has four groups: susceptible (S), exposed (E), infectious (I), and recovered (R), with the total population size *N* = *S*+*E*+*I*+*R*. For ILI, the immunity of individuals after recovery is only temporary, and there is still the possibility of reinfection. We emphasized that both infected persons and exposed persons who may be in the incubation period were infectious. Meanwhile, this study did not consider dramatic population changes during only a few years. Therefore, the SEIRS model was adopted in this study to describe the transmission of ILI. The specific equation of the SEIRS model adopted was as follows:


(6) $$\left\{ \begin{array}{l} \frac{dS}{dt}=-\frac{r\beta IS}{N}-\frac{r_{2}\beta_{2}ES}{N}+\lambda R\\ \frac{dE}{dt}=\frac{r\beta IS}{N}-\alpha E+\frac{r_{2}\beta_{2}ES}{N}\\ \frac{dI}{dt}=\alpha E-\gamma I\\ \frac{dR}{dt}=\gamma I-\lambda R \end{array}\right.$$

In Equation (6), the *N* represents the total number of people in the region. *S, E, I*, and *R* denote susceptible, exposed, infected, and recovered group, respectively. For each unknown parameter, β is the probability of the infected person transmitting the virus to the susceptible person, β_2_ is the probability of the exposed person transmitting the virus to the susceptible person, *r* denotes the average number of susceptible persons in contact with infected persons, *r*_2_ denotes the average number of susceptible persons in contact with exposed persons, α represents the probability of the exposed person becoming infected, γ represents the probability of the infected person recovering to health, and λ indicates the probability of the recovered person returning to a susceptible state. The optimal estimates of these parameters were estimated by the historical ILI% data of each city based on the minimum fitted error.

#### The LSTM Method

The LSTM neural network is an improved algorithm based on the recurrent neural network (RNN). By adding a “gate” structure, it overcomes the shortcomings of RNN and avoids the vanishing gradient problem. “Gate” structure can selectively forget or memorize information, including the existence of a sigmoid activation function. A LSTM unit contains three gates: forgetting gate, input gate, and output gate, which makes the invariable self-cycling weight matrix in RNN change. These changed weights are adjusted continuously according to the learning process, so as to solve the problem of gradient disappearance.

### Performance Validation

The performance validation step included residual checking and performance comparison.

For the residual checking part, since a well-behaved VARMA model should ideally extract all the regular pattern information out of the original data, the residuals of the model were white noise (i.e., the data should be completely random series without any regular pattern information). Therefore, the Ljung-Box statistics was utilized in this study to verify the model by testing whether residuals of the model were only white noises.

As for the performance comparison part, we compared the results of the STN with those of the other two types of models. To this end, the accuracy of the methods was measured. The accuracy evaluated whether the forecasted values of the ILI% were close to the real ones. In this study, considering that the value of ILI% was decimal, the relative measurement index would be appropriate to distinguish the difference of forecasting performance of the models, so we adopted mean absolute percentage error (MAPE) to monitor the accuracy. In addition, to assess the forecasting performance of the model, the whole time series data was split into the training set and the testing set. We used the first set for the test of model fitting and the second one for forecasting. For the overall performance, the training set integrated the data from the 1st week of 2010 to the 26th week of 2017, while the testing set data consisted of data from the second-half year of 2017. Furthermore, we also tried several different dataset splitting methods (see Section Seasonal Forecast) to evaluate the seasonal forecasting performance.

### Seasonal Forecast

Influenza has the characteristic of seasonal epidemics, and the high incidence season of disease epidemics is from November to March of the next year. In order to study whether there was a difference in the forecasting performance of STN in the high incidence season and the low incidence season of the disease, and the robustness of the forecasting performance of STN, the last high incidence season (data integrated from the 44th week of 2016 to the 13th week of 2017) and the last low incidence season (data integrated from the 14th week of 2017 to the 43rd week of 2017) were specifically divided from the original data set for forecasting verification. The forecasting performances of the STN and the other two models were compared in the high and low incidence seasons of ILI.

All the above statistical tests were conducted at the statistically significant level of 0.05. The SKATER was performed in GeoDa 1.18.0.0. SEIRS model and LSTM model were performed in MATLAB R2021a. The rest of the statistical analyses were performed in R 4.0.1 using the computing packages {MTS} for the VARMA model and {G1DBN} for DBN.

## Results

### Cluster Partitioning

**Figure 1:**
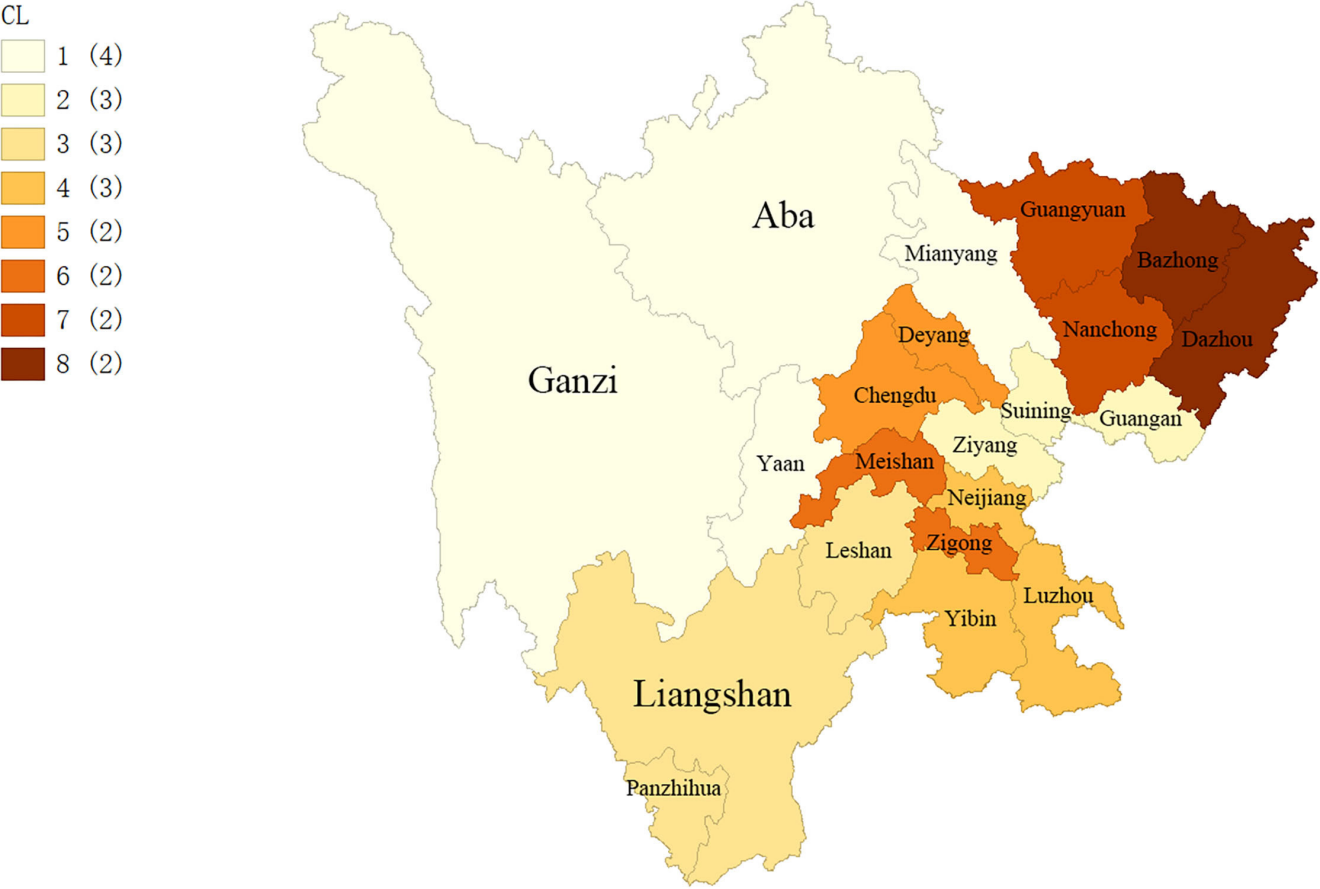
The cluster results of spatial kluster analysis by tree edge removal (SKATER) algorithm.

The grouping of eight clusters based on the SKATER algorithm is shown in Figure 1. However, this study did not include the clusters where these cities may contain outliers. Therefore, 14 cities in Sichuan Province were included, which were divided into 5 clusters (i.e., cluster 1: Aba, Mianyang, Ganzi, and Ya'an; cluster 2: Guang'an, Suining, and Ziyang; cluster 3: Yibin, Luzhou, and Neijiang; cluster 4: Chengdu and Deyang; cluster 5: Meishan and Zigong).

### Data Preparation and Description

**Figure 2:**
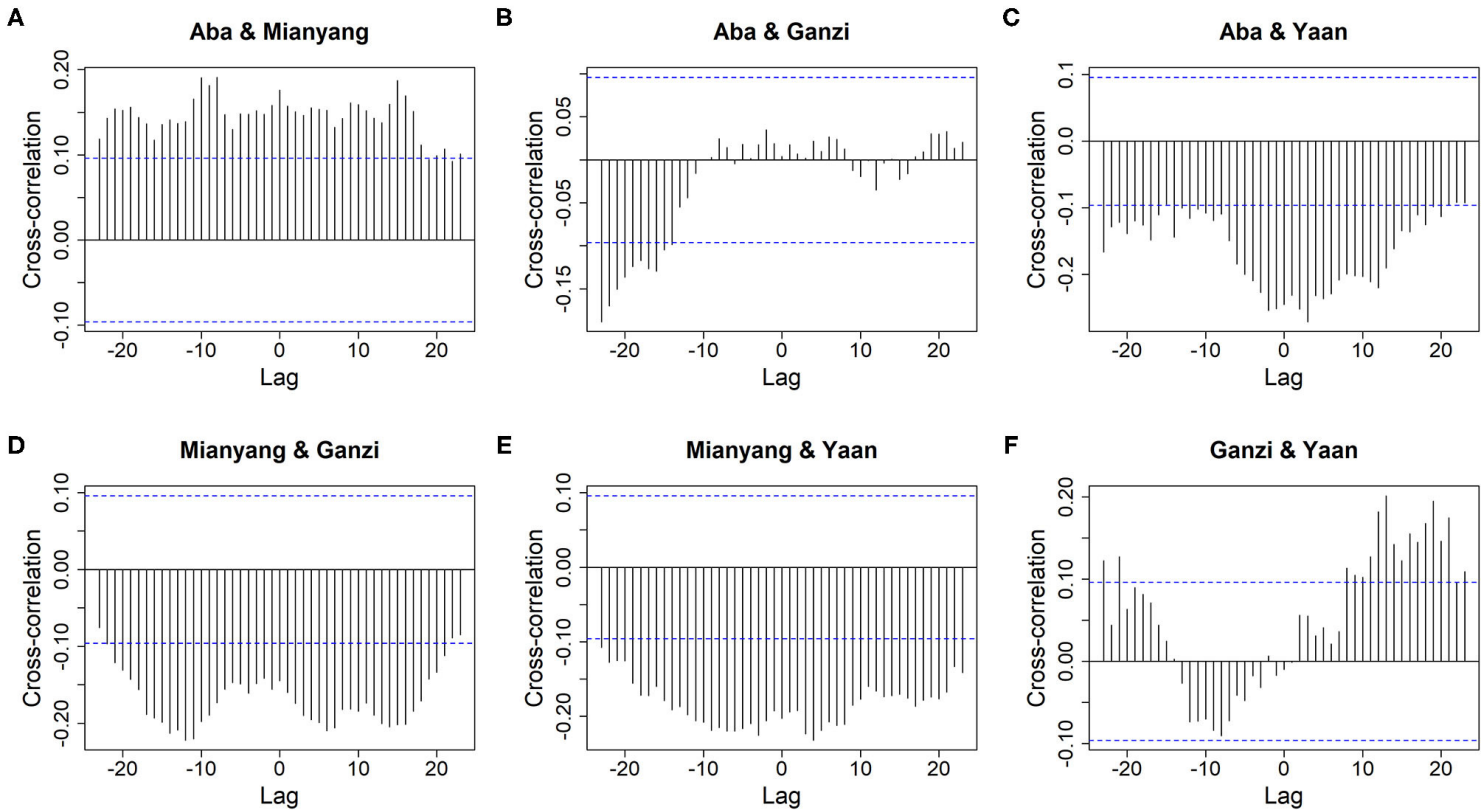
The cross-correlation of ILI% between cities of cluster 1. **(A)** Aba and Mianyang. **(B)** Aba and Ganzi. **(C)** Aba and Ya'an. **(D)** Mianyang and Ganzi. **(E)** Mianyang and Ya'an. **(F)** Ganzi and Ya'an.

**Figure 3:**
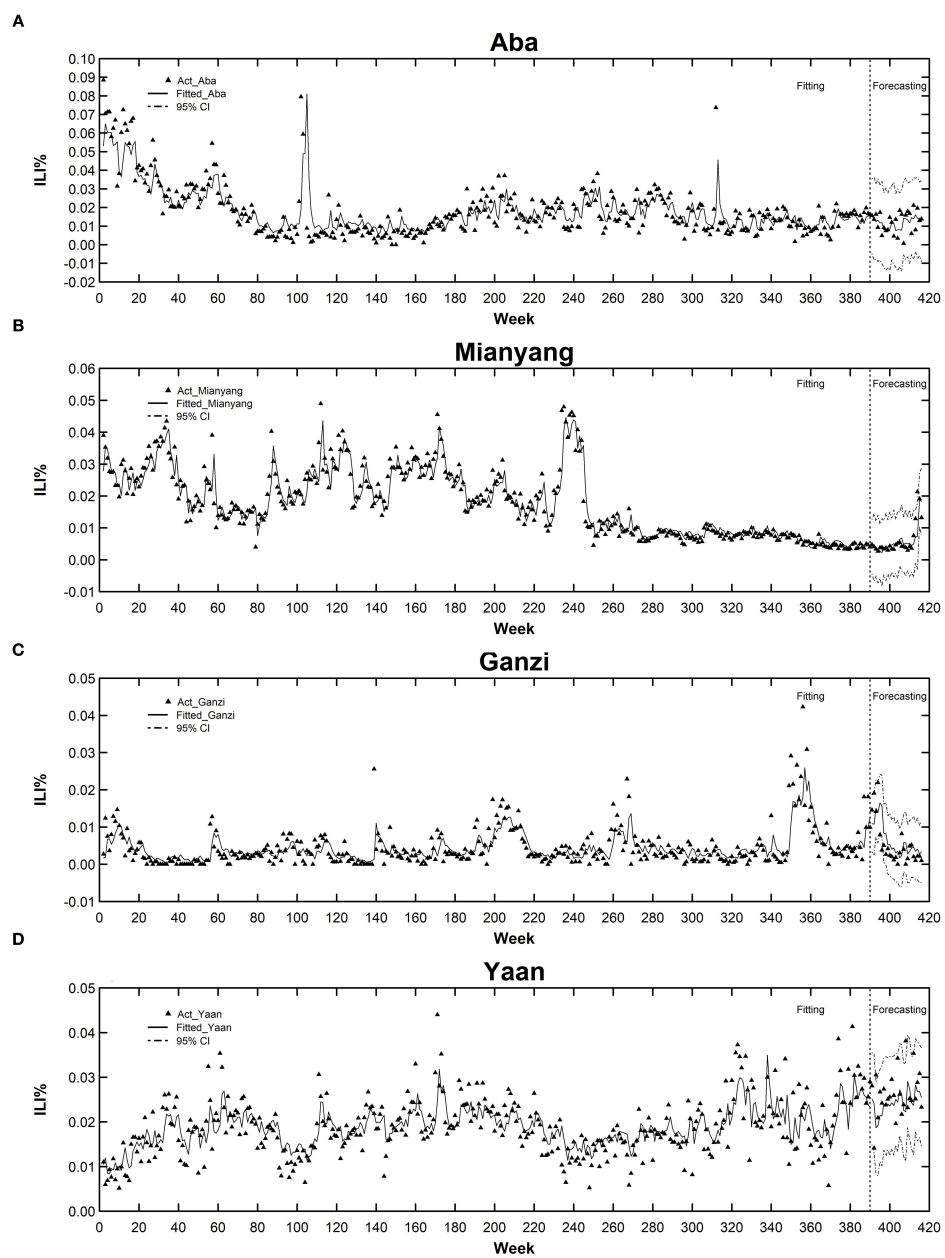
The fitted/forecasted time series vs. the actual values for cities of cluster 1. **(A)** Aba. **(B)** Mianyang. **(C)** Ganzi. **(D)** Ya'an.

The ILI% series of actual values of the 14 cities in Sichuan could be seen from **Figure 3** and Supplementary Figures 1–4. In most time series, stationarity could be visually verified since there was no dramatic change. Furthermore, except for Chengdu, Suining, and Ziyang, the unit-root test showed that the values of *P* of other cities were less than 0.05, which further confirmed that the time series were stationary. For Chengdu, Suining, and Ziyang, cointegration tests were performed on ILI% time series vectors of all cities in their clusters (i.e., clusters 2 and 4). The results were significant, indicating that there were both cointegrations among ILI% sequences of cities in these two clusters. Besides, from the cross-correlation plots shown in Figures 2A–F and Supplementary Figures 5–8, the existence of dynamic correlations among the ILI% time series had been verified. Therefore, it was appropriate to use the VARMA model for further analysis.

### Estimates of the VARMA Model

In this study, we estimated the VARMA model for 1–390 weeks. The best model for each cluster is listed in Table 1.

**Table 1:** The best vector autoregressive moving average (VARMA) model for each cluster.

**Cluster**	**Kronecker index**	**VARMA model**
1	(1, 1, 1, 1)	(1, 1)
2	(1, 1, 1)	(1, 1)
3	(2, 1, 1)	(2, 2)
4	(1, 1)	(1, 1)
5	(1, 1)	(1, 1)

### Performance Validation

It could be seen from Figure 3 and Supplementary Figures 1–4 that both the fitted values and forecasted values approximated very well to the actual ones. In addition, the forecasting part also exhibited that the 95% *CI* of forecasted values could basically contain the actual values, which suggested the STN was also good at precision. More details about the residual checking and model comparison are given below.

#### Residual Checking

**Figure 4:**
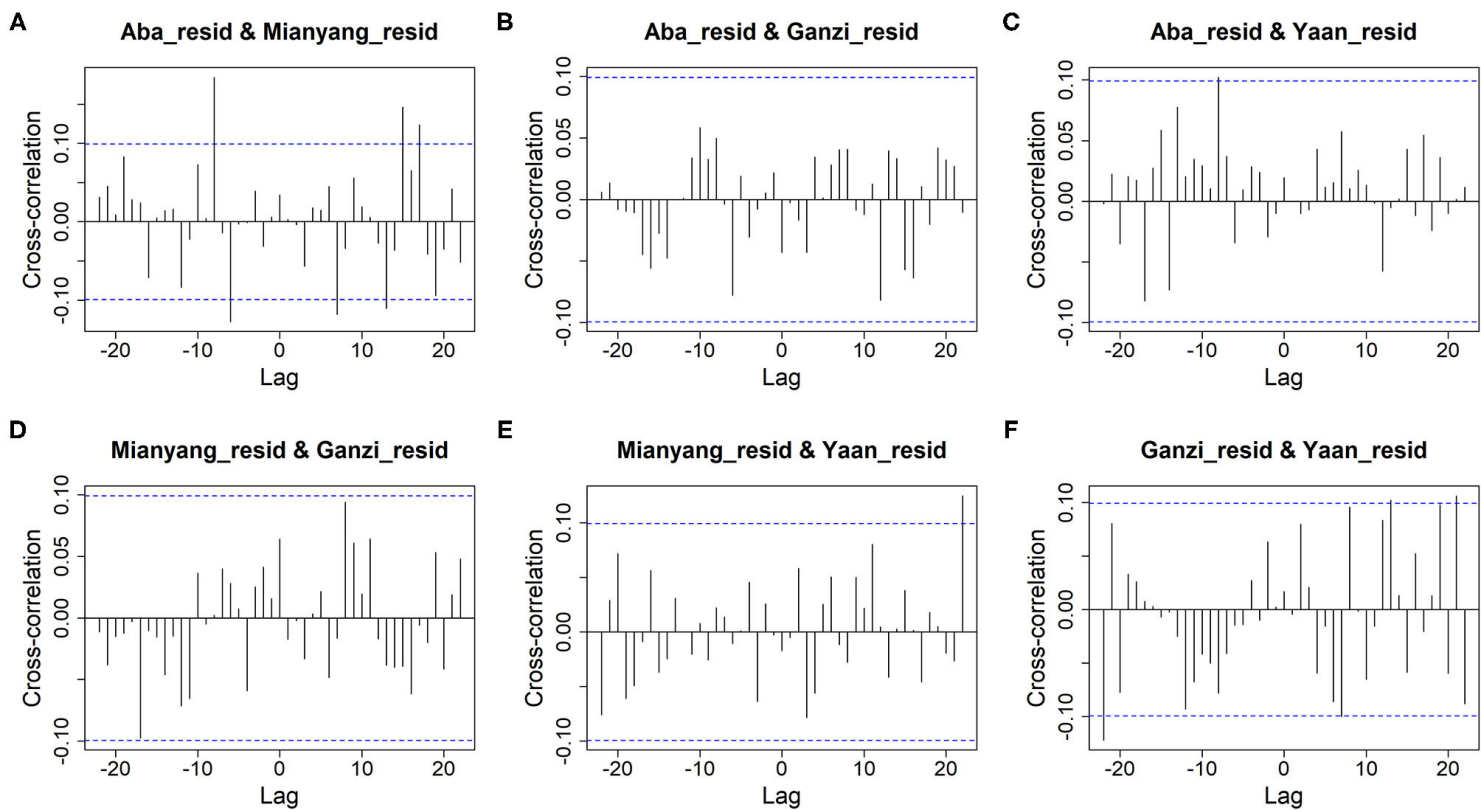
The cross-correlation of the residuals between cities of cluster 1. **(A)** The residuals of Aba and Mianyang. **(B)** The residuals of Aba and Ganzi. **(C)** The residuals of Aba and Ya'an. **(D)** The residuals of Mianyang and Ganzi. **(E)** The residuals of Mianyang and Ya'an. **(F)** The residuals of Ganzi and Ya'an.

For cluster 1, the *Q* statistics for 1-, 5-, 10-, and 20-lag Ljung-Box tests were 0.56 (*df* = 16, *P* = 1.00), 45.84 (*df* = 80, *P* = 1.00), 136.76 (*df* = 160, *P* = 0.91), and 294.01 (*df* = 320, *P* = 0.85), respectively, which validated that the VARMA model had fully extracted the useful information out of the original data and that the left residuals were only white noise series. Meanwhile, compared with Figures 2A–F, 4 displays that the information of cross-correlation between dynamic time series of cluster 1 has been fully extracted by the VARMA model. The other clusters had similar results. More details are given in Supplementary Figures 9–12.

#### Performance Comparisons

Comparing the forecasting accuracy of STN, SEIRS, and LSTM models, Table 2 lists the MAPE value comparison of the three models. The average MAPE of the 14 cites of STN was 31.134, which was 25.26 and 49.82% less than that of the LSTM and SEIRS respectively. At the city level, the MAPE of STN was almost the smallest among the three models, with only a few exceptions. For example, the forecasting accuracy of STN was not the best among the three models in the three cities in cluster 2, which might be affected by the instability of the original data series of Suining and Ziyang. Although there was a cointegration relationship between the three sequences in this cluster, the instability of the sequence still affected the forecasting performance of the STN model. In addition, for the forecasting of Luzhou, SEIRS performed slightly better than STN, which might be influenced by the geographical location of Luzhou. As Luzhou is located at the intersection of Sichuan, Yunnan, Guizhou, and Chongqing, its epidemic situation is likely to be affected by other cities outside the province, which was out the scope of this study since it only built networks within Sichuan Province. However, even in this case, the forecasting performance of the other two cities in cluster 3 was still good. As for Ganzi, it could be seen from Figure 3 that ILI% remains at a low level from 2010 to 2015, and the incidence of ILI in some weeks was reported to be zero. Except for the above plausible exceptions, the accuracy of STN forecasting was generally better than that of the SEIRS and LSTM models.

**Table 2:** The comparison of the forecasting mean absolute percentage error (MAPE) of the three methods.

**Cluster**	**City**	**STN**	**LSTM**	**SEIRS**
1	Aba	112.875	158.754	147.466
	Mianyang	27.797	51.749	81.770
	Ganzi	101.340	49.793	80.979
	Ya'an	17.298	19.708	89.588
2	Guang'an	9.329	6.182	40.684
	Suining	22.778	15.485	36.475
	Ziyang	15.834	24.119	13.515
3	Yibin	14.192	54.143	73.715
	Luzhou	23.741	60.174	22.218
	Neijiang	16.061	28.275	40.692
4	Chengdu	16.756	20.194	50.962
	Deyang	31.440	40.642	77.262
5	Meishan	14.886	24.695	59.439
	Zigong	11.550	29.290	53.774
AVERAGE		31.134	41.657	62.039

*The minimum MAPE value of the three methods for each city is indicated in bold and underlined in the table*.

### The Results of the Seasonal Forecast

#### The Forecast of High Incidence Season

The training set and testing set were redivided, and the forecast analysis of high incidence season used data integrated from the 1st week of 2010 to the 43rd week of 2016 as the training set, data integrated from the 44th week of 2016 to the 13th week of 2017 as the testing set. Table 3 lists the comparison of MAPE values of the three models in forecast analysis in high incidence season. According to the mean MAPE of the 14 cities, the forecasting accuracy advantage of STN was more obvious than that of LSTM and SEIRS. The MAPE value of STN decreased to 24.742 when compared with that before the seasonal division of the forecast set, and the MAPE value of LSTM reached about three times that of STN. For each city except Zigong, the accuracy of STN forecasting of the other cities was better than that of the other two models. In Zigong, the forecasting accuracy of LSTM was slightly better than that of STN. However, it did not affect the overall assessment of performance since the MAPE in Zigong was already very low and the difference between STN and LSTM was even lower.

**Table 3:** The comparison of the forecasting MAPE in the high incidence season of the three methods.

**Cluster**	**City**	**STN**	**LSTM**	**SEIRS**
1	Aba	64.262	74.410	119.943
	Mianyang	36.846	60.975	81.193
	Ganzi	45.376	55.637	89.901
	Ya'an	35.458	37.202	86.989
2	Guang'an	9.474	21.776	44.346
	Suining	10.518	28.262	50.281
	Ziyang	20.861	36.777	66.618
3	Yibin	12.189	84.445	59.409
	Luzhou	25.449	32.391	31.811
	Neijiang	17.640	46.750	19.606
4	Chengdu	24.027	27.664	48.388
	Deyang	16.326	71.065	69.006
5	Meishan	16.441	68.871	23.672
	Zigong	11.521	9.332	47.470
AVERAGE		24.742	46.826	59.902

*The minimum MAPE value of the three methods for each city is indicated in bold and underlined in the table*.

#### The Forecast of Low Incidence Season

In the forecasting analysis of low incidence season, we included the data integrated from the 1st week of 2010 to the 13th week of 2017 as the training set, and the data integrated from the 14th week of 2017 to the 43rd week of 2017 as the testing set. Table 4 lists the comparison of MAPE values of the three models in the forecasting analysis of low incidence season. According to the mean MAPE of the 14 cities, the forecasting MAPE of STN decreased to 26.209 when compared with that before the seasonal division of the forecast set and was still better than that of LSTM and SEIRS. At the city level, the accuracy of STN was better than that of the other two models for most cities excluding Mianyang, Ganzi, and Ziyang. The low accuracy of STN forecasting in Ganzi and Ziyang might be owed to the reasons mentioned in Section Performance comparisons, and the accuracy of Ganzi affected that of Mianyang since they were in the same cluster.

**Table 4:** The comparison of the forecasting MAPE in the low incidence season of the three methods.

**Cluster**	**City**	**STN**	**LSTM**	**SEIRS**
1	Aba	89.490	115.434	121.485
	Mianyang	22.858	15.868	51.397
	Ganzi	78.919	63.378	86.207
	Ya'an	18.395	23.174	89.810
2	Guang'an	12.344	15.428	52.549
	Suining	16.127	17.211	42.276
	Ziyang	10.869	10.305	11.566
3	Yibin	15.823	35.166	70.253
	Luzhou	20.373	39.322	21.194
	Neijiang	17.539	40.347	38.066
4	Chengdu	12.047	32.142	39.873
	Deyang	25.111	35.297	71.895
5	Meishan	15.565	28.213	51.848
	Zigong	11.463	21.537	57.054
AVERAGE		26.209	35.202	57.534

*The minimum MAPE value of the three methods for each city is indicated in bold and underlined in the table*.

In general, the accuracy of STN forecasting in both high and low incidence seasons was the best among the three models, and the MAPE value of STN decreased significantly when compared with the forecast set which was not divided according to the seasonality of influenza. To be more specific, the numbers of regions with the best accuracy of STN forecasting in high incidence season and low incidence season increased, especially in high incidence season. This showed that STN had good forecasting performance and strong robustness in both high and low incidence seasons. Furthermore, STN had better forecasting accuracy either in the high or low incidence season, but it was more suitable in the high incidence season.

## Discussion

The results of this study illustrated that the STN had advantages in forecasting performance of infectious diseases across multiple regions. The accuracy of its forecasting performance was superior to SEIRS and LSTM. Combined with previous research results (6), it could be seen that the STN would be very helpful in guiding the practical prevention and control work of infectious diseases.

According to Stoto (28), a practical surveillance system should include three parts: continuous monitoring of multivariate data, applying algorithms to raise the alarm when something unusual is happening, and a protocol on how to respond to an alarm. It was observed from our results that the STN could assist to improve all three parts of the surveillance system. First, as a multivariate time series analysis model, the STN model could inherently integrate and extract information from multivariate data, which could not only be limited to a single variable (e.g., ILI%) across regions but could also contain other types of variables (e.g., meteorological, social-economic, and environmental factors). The second advantage of STN was that its results could suggest when and where the next outbreak would probably occur and how serious the next outbreak would be, so that it could provide suggestions and evidence on whether an alarm should be raised. Last but not least, the information of direction and time-lag of influenza transmission exhibited by STN could also indicate the way about how to respond to an alarm. For example, this study found that there was a 1-week lagging transmission route from Yibin to Luzhou (the estimated effect was 0.248), which suggested that Luzhou should pay attention to the epidemic situation of Yibin. The same suggestion was also applicable to Yibin, where there was a 2-week lagging transmission route from Neijiang to Yibin (the estimated effect was 0.014). Furthermore, the lag effect as long as 2 weeks also reminded that the surveillance of influenza should maintain a long enough temporal window for early warning. Since the interpretation of the STN model and its potential application were highly consistent with the establishment of a multi-point trigger and a multi-channel surveillance mechanism of infectious diseases (29), it was expected that the STN model could probably serve as a new tool for the improvement of intelligent early warning of infectious diseases.

Except for the interpretation of results, there were still other features that could also guarantee the potential use of STN in the practice of influenza surveillance.

First, the VARMA model could provide evidence of both strengths of association and temporality, which were the two key points of the Hill's criteria for causality (30). Meanwhile, under some mild conditions, the probability distribution of the VARMA model could be reliably represented as a causal network, and the latter was a commonly used tool in causal inference (24). To this end, it was plausible that the STN could at least partly serve for the etiological study to screen crucial clues of the cause-and-effect relationships.

Secondly, from the perspective of representativeness, China is a large country with great diversities among different areas in many aspects (e.g., environment, population, economy, and social customs), and Sichuan Province has the fourth largest population, the fifth largest land, and the sixth highest GDP in China. Besides, the population in Sichuan province includes 55 of China's 56 ethnic groups, and there are many major types of landforms in Sichuan (e.g., mountains, hills, plateaus, and plains). Therefore, the application of STN to the surveillance of influenza in Sichuan province suggested that it could also be considered for infectious diseases surveillance in other areas.

However, it should also be acknowledged that there were still some limits in our study. For example, this study provided our explanations based on the features of our analyzed results and some practical experience, so relevant etiological and laboratory data were still in need to further identify the speculations about the spreading direction of influenza in the Sichuan province. Besides, there was a long way to go from model building to making causal inferences about influenza epidemics in the real world. Therefore, it was highly expected that the STN could provide a new way for causal inference in the surveillance of infectious diseases.

## Conclusion

This study applied the STN to forecasting infectious diseases across multiple regions. The results illustrated that the STN not only had good accuracy in forecasting performance but could also indicate the spreading directions of infectious diseases among multiple regions to a certain extent. Therefore, the STN was a promising candidate to improve the surveillance work.

## Data Availability Statement

The data analyzed in this study is subject to the following licenses/restrictions: the data that support the findings of this study are available from Sichuan Center for Disease Control and Prevention but restrictions apply to the availability of these data, which were used under license for the current study, and so are not publicly available. Data are however available from the authors upon reasonable request and with permission of Sichuan Center for Disease Control and Prevention. Requests to access these datasets should be directed to CY, changhong_yang@163.com.

## Author Contributions

HWang consulted the literature, analyzed the data, and was a major contributor in writing the manuscript. JQ analyzed the data and wrote part of the manuscript. CL wrote part of the manuscript and checked the full manuscript. HWan analyzed the results. CY collected the data. TZ analyzed the data and reviewed all the material. All authors contributed to the article and approved the submitted version.

## Funding

This research work was funded by Sichuan Science and Technology Program (2020YFS0015, 2020YFS0091, and 2021YFS0001-LH), Health Commission of Sichuan Province (20PJ092), National Natural Science Foundation of China (81602935), Chongqing Science and Technology Program (cstc2020jscxcylhX0003), Chengdu Science and Technology Program (2021-YF05-01585-SN), Sichuan University (2018hhf-26), Central government funding items (2021zc02), and Liangshan Prefecture Center for Disease Control and Prevention (H210322).

## Conflict of Interest

The authors declare that the research was conducted in the absence of any commercial or financial relationships that could be construed as a potential conflict of interest.

## Publisher's Note

All claims expressed in this article are solely those of the authors and do not necessarily represent those of their affiliated organizations, or those of the publisher, the editors and the reviewers. Any product that may be evaluated in this article, or claim that may be made by its manufacturer, is not guaranteed or endorsed by the publisher.
