# Supplementary material for: Applying the Spatial Transmission Network to the Forecast of Infectious Diseases Across Multiple Regions
Source: Front Public Health. 2022 Mar 11;10:774984. doi: 10.3389/fpubh.2022.774984 (PMC8962516; doi:10.3389/fpubh.2022.774984)
Supplement: Supplementary file 1 [file Data_Sheet_1.pdf]

Supplementary Material

1 The fitted/forecasted time series vs. the actual values

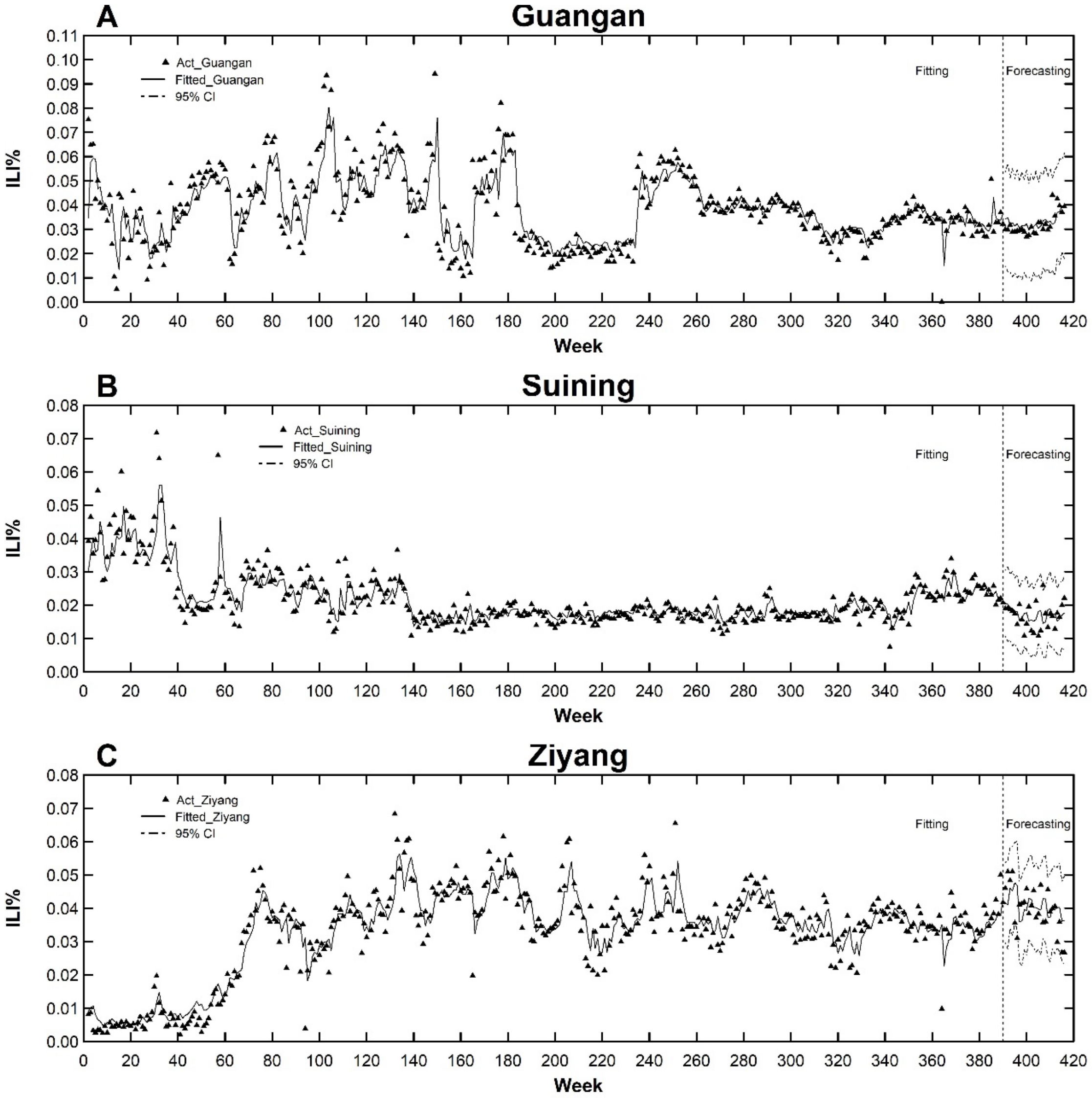

Supplementary Figure 1. The fitted/forecasted time series vs. the actual values for cities of cluster 2. **(A)** Guang'an; **(B)** Suining; **(C)** Ziyang.

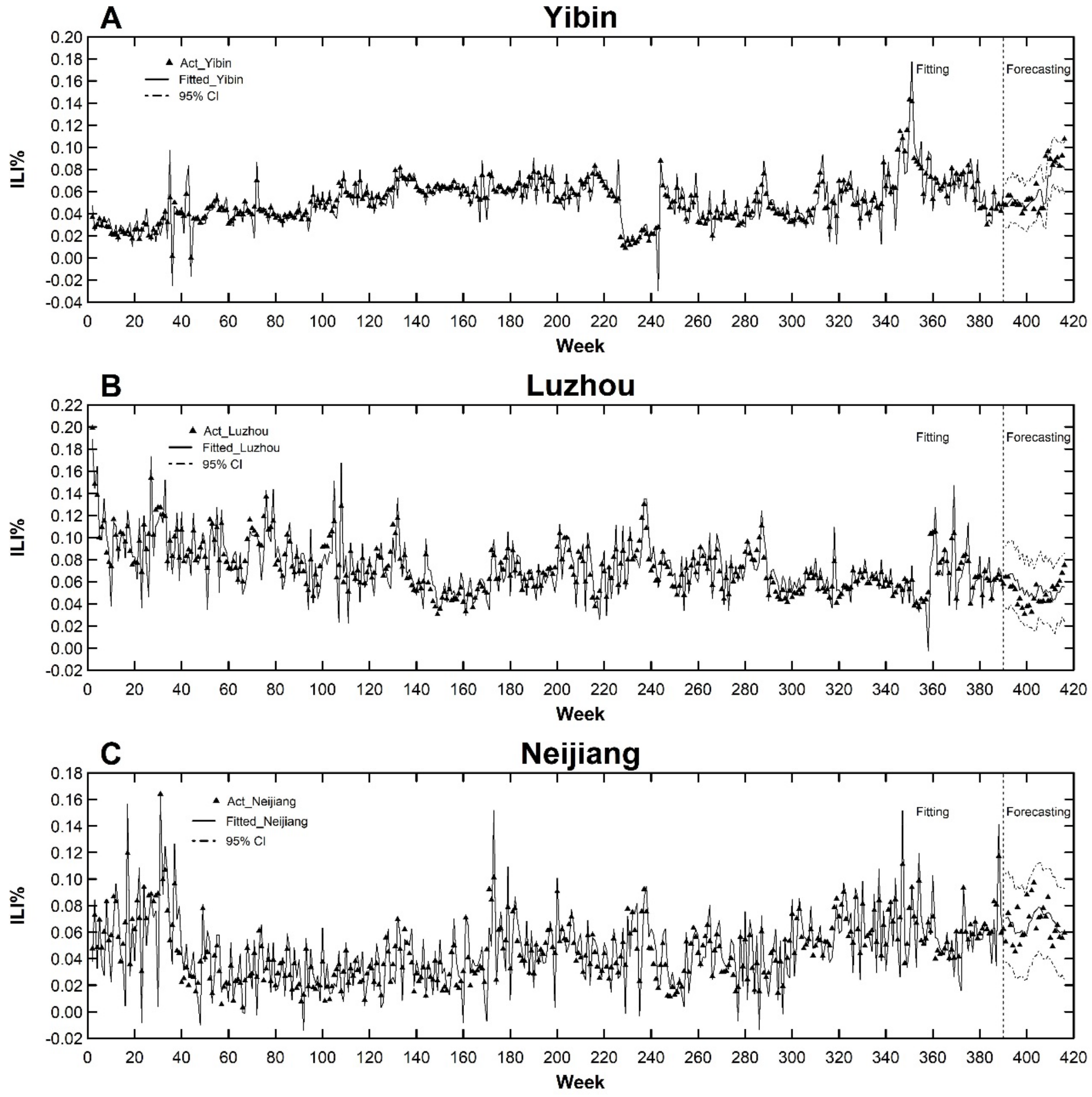

Supplementary Figure 2. The fitted/forecasted time series vs. the actual values for cities of cluster 3. **(A)** Yibin; **(B)** Luzhou; **(C)** Neijiang.

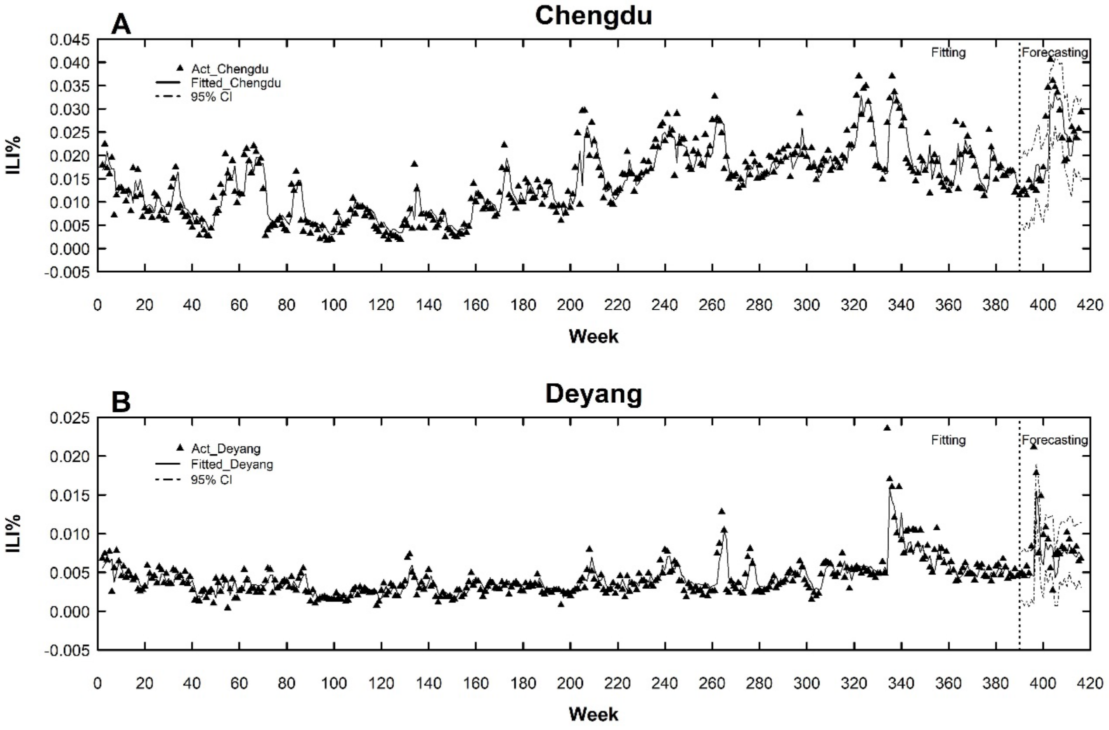

Supplementary Figure 3. The fitted/forecasted time series vs. the actual values for cities of cluster 4. **(A)** Chengdu; **(B)** Deyang.

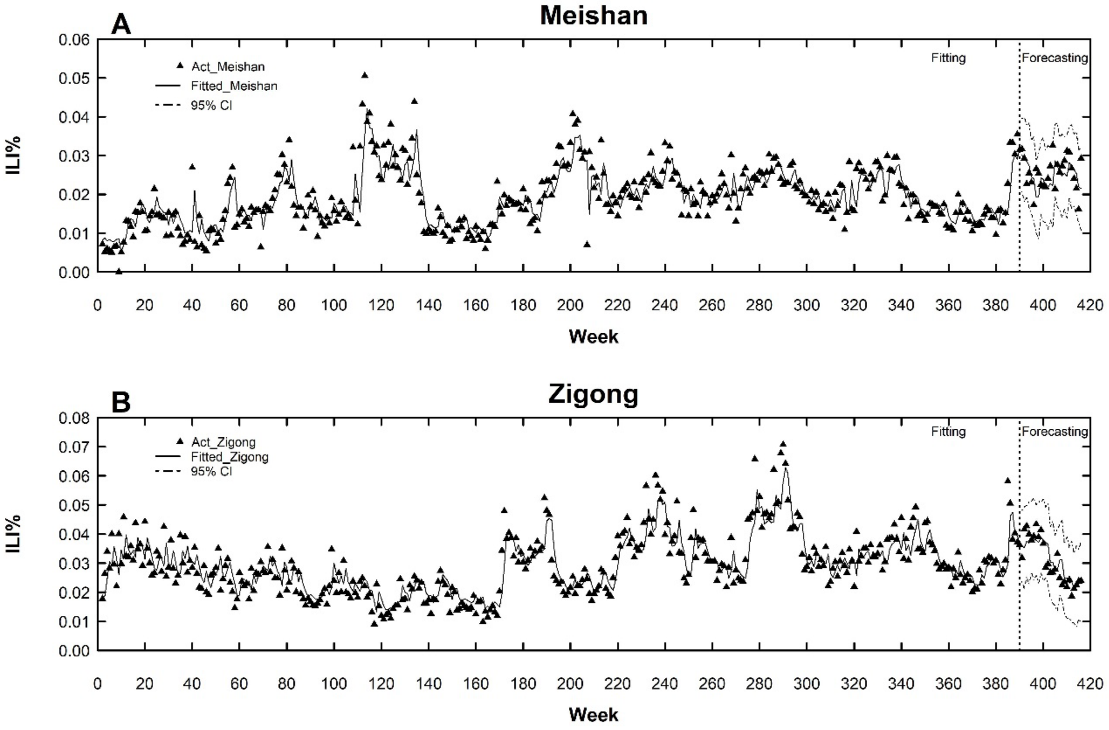

Supplementary Figure 4. The fitted/forecasted time series vs. the actual values for cities of cluster 5. **(A)** Meishan; **(B)** Zigong.

2 The cross-correlation of ILI% between cities

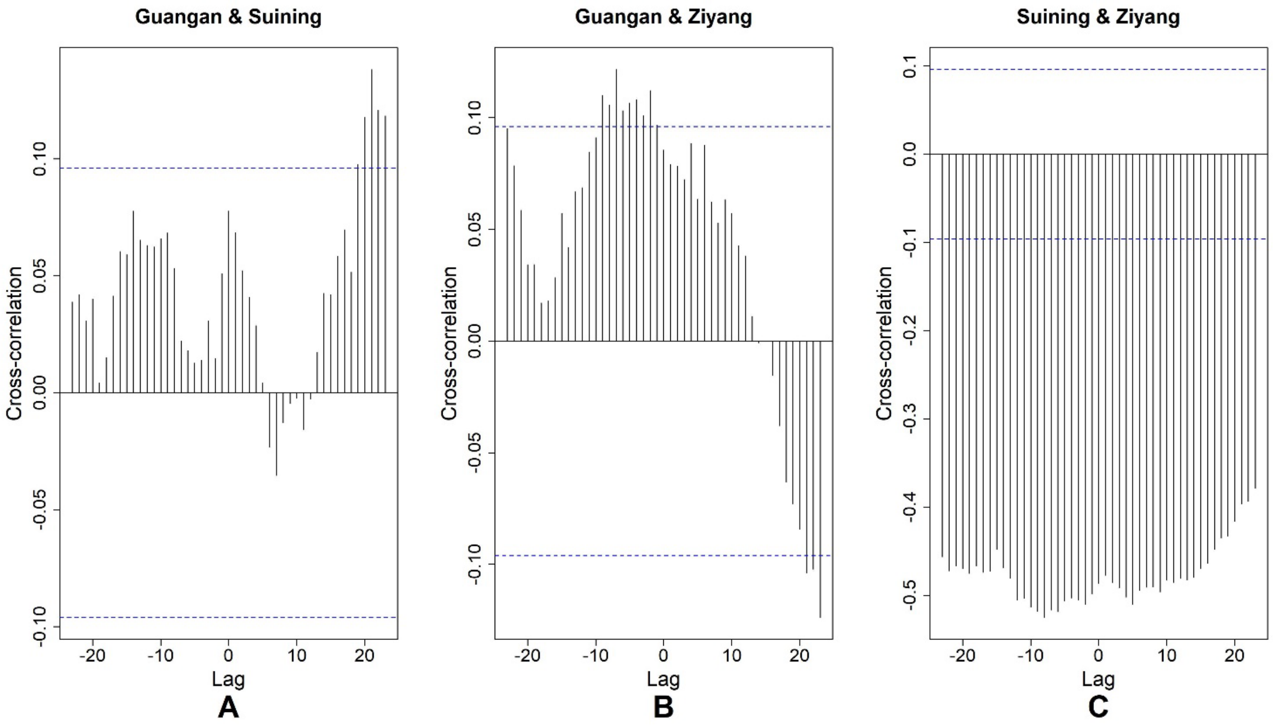

Supplementary Figure 5. The cross-correlation of ILI% between cities of cluster 2. **(A)** Guang'an & Suining; **(B)** Guang'an & Ziyang; **(C)** Suining & Ziyang.

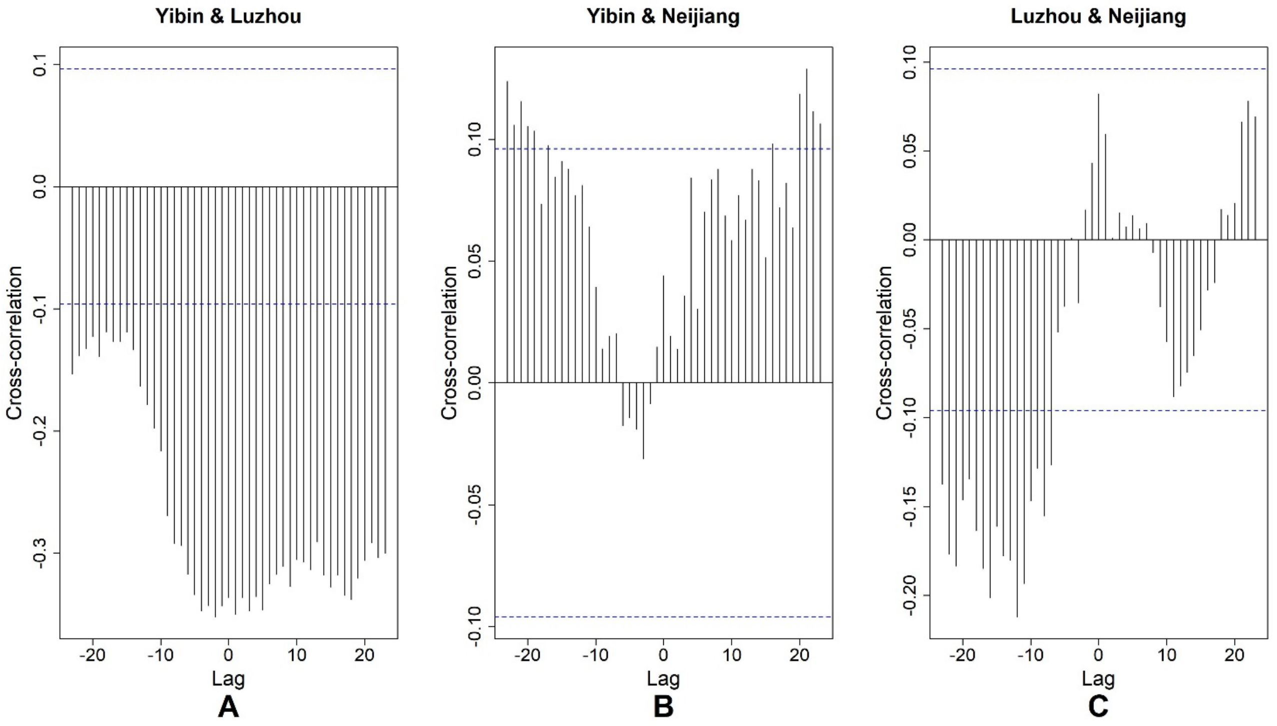

Supplementary Figure 6. The cross-correlation of ILI% between cities of cluster 3. **(A)** Yibin & Luzhou; **(B)** Yibin & Neijiang; **(C)** Luzhou & Neijiang.

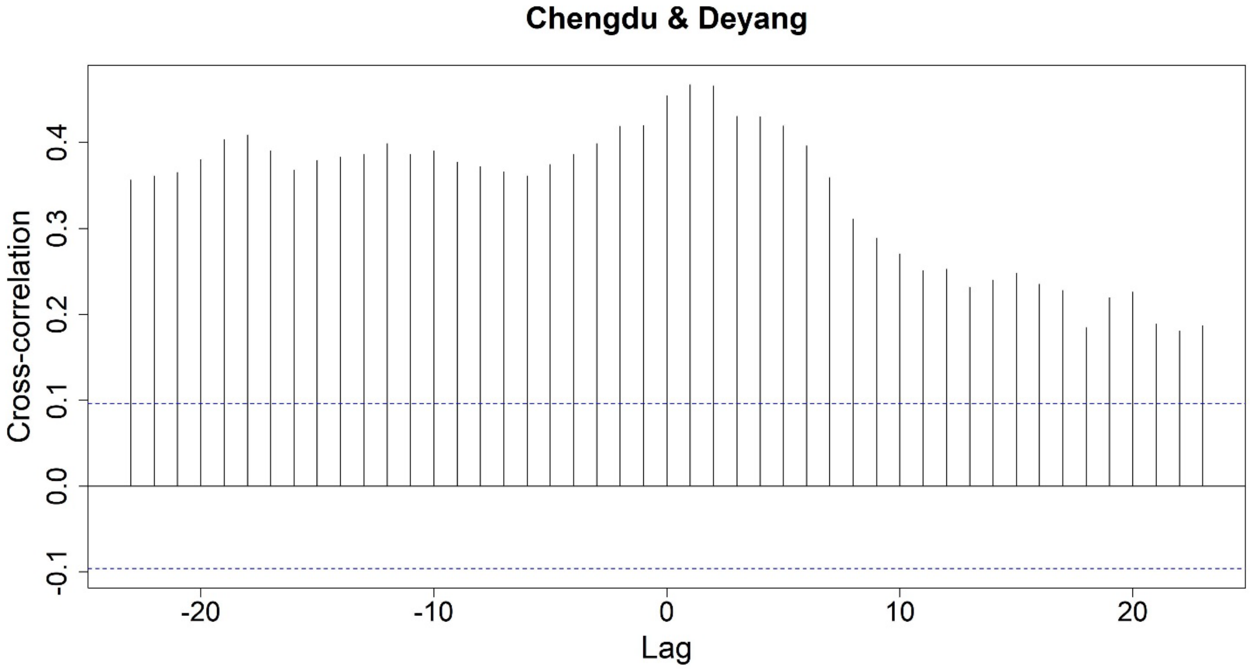

Supplementary Figure 7. The cross-correlation of ILI% between cities of cluster 4.

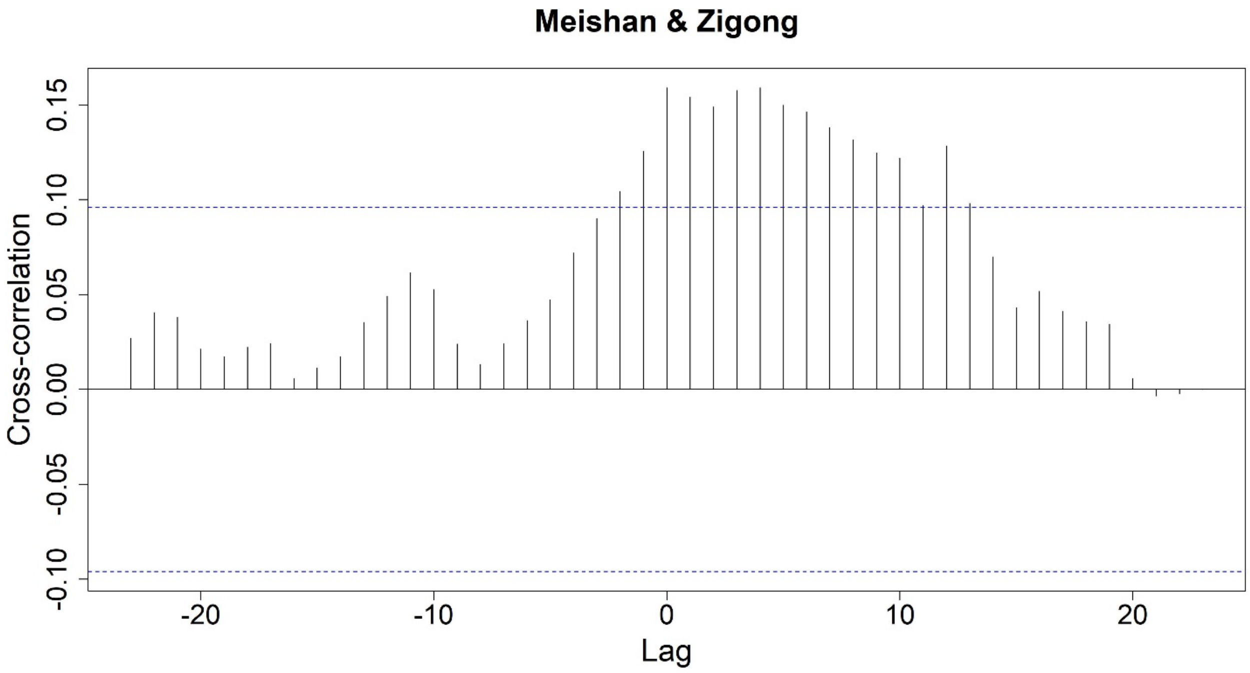

Supplementary Figure 8. The cross-correlation of ILI% between cities of cluster 5.

3 The cross-correlation of the residuals between cities

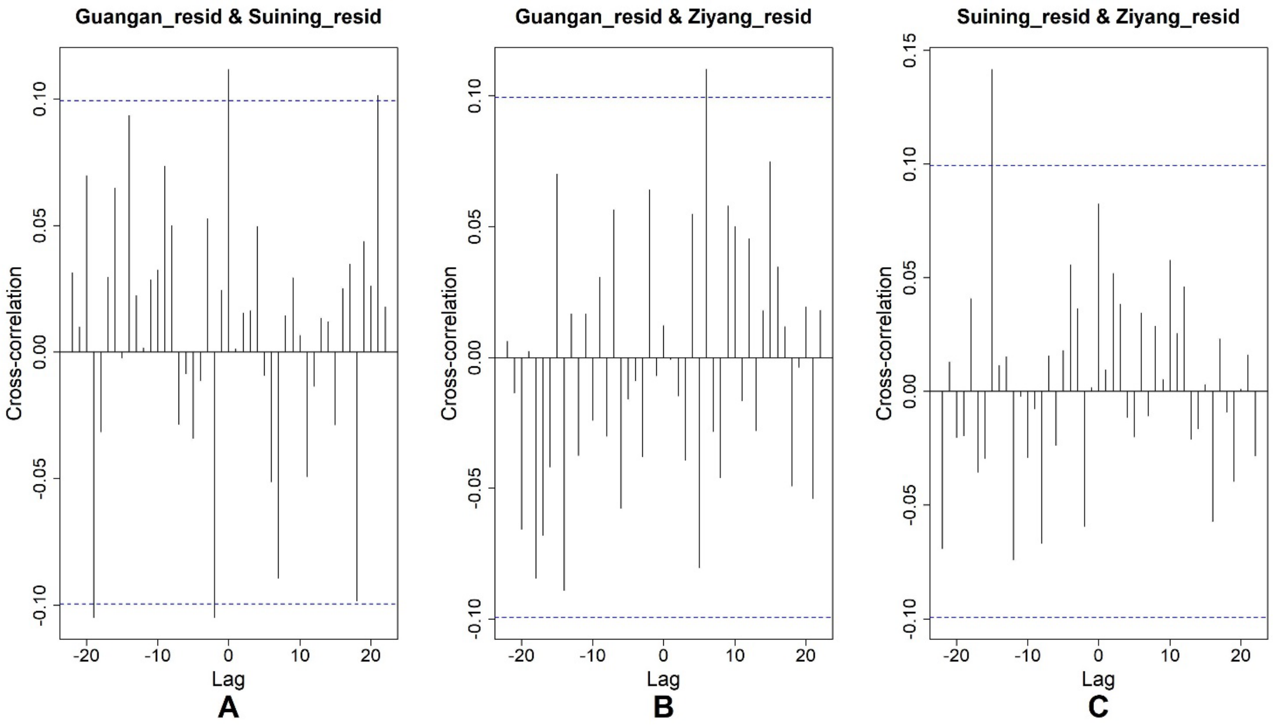

Supplementary Figure 9. The cross-correlation of the residuals between cities of cluster 2. **(A)** The residuals of Guang'an & Suining; **(B)** The residuals of Guang'an & Ziyang; **(C)** The residuals of Suining & Ziyang.

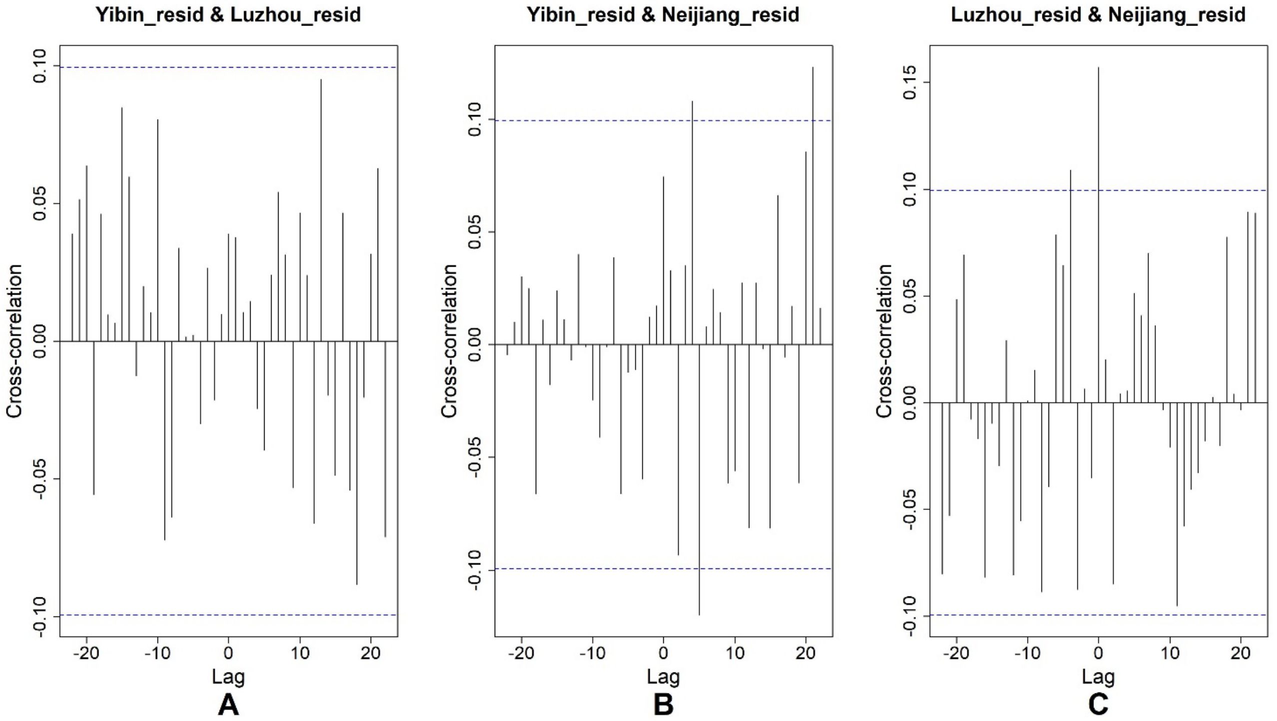

Supplementary Figure 10. The cross-correlation of the residuals between cities of cluster 3. **(A)** The residuals of Yibin & Luzhou; **(B)** The residuals of Yibin & Neijiang; **(C)** The residuals of Luzhou & Neijiang.

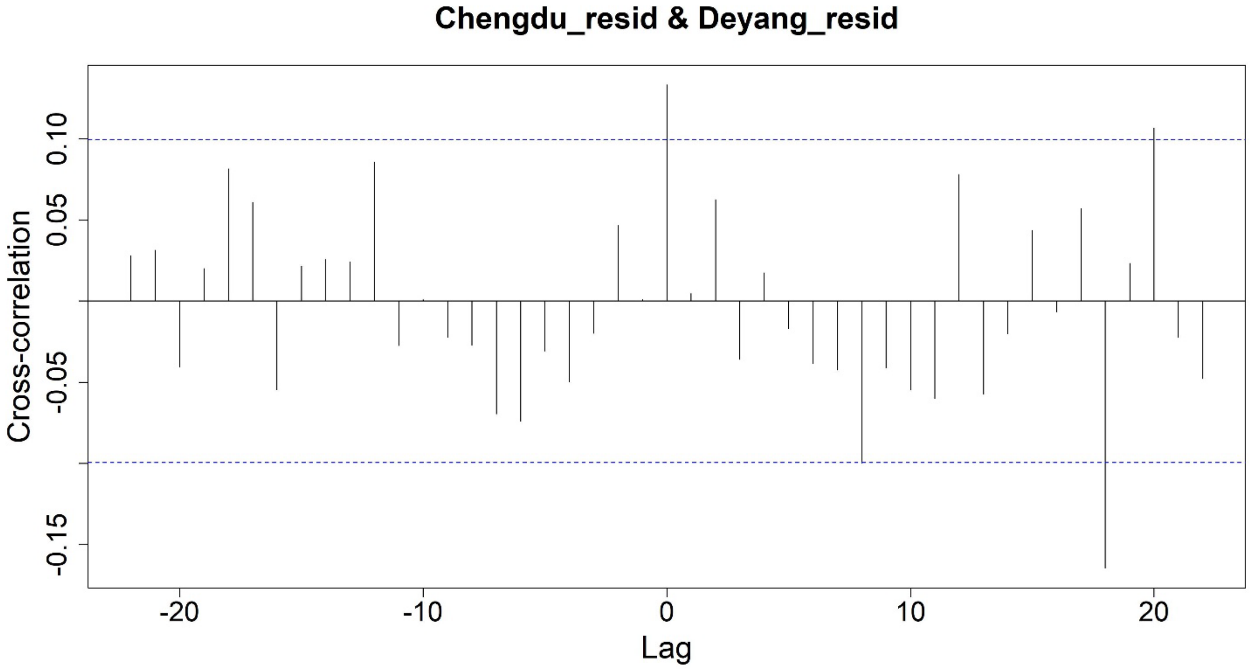

Supplementary Figure 11. The cross-correlation of the residuals between cities of cluster 4.

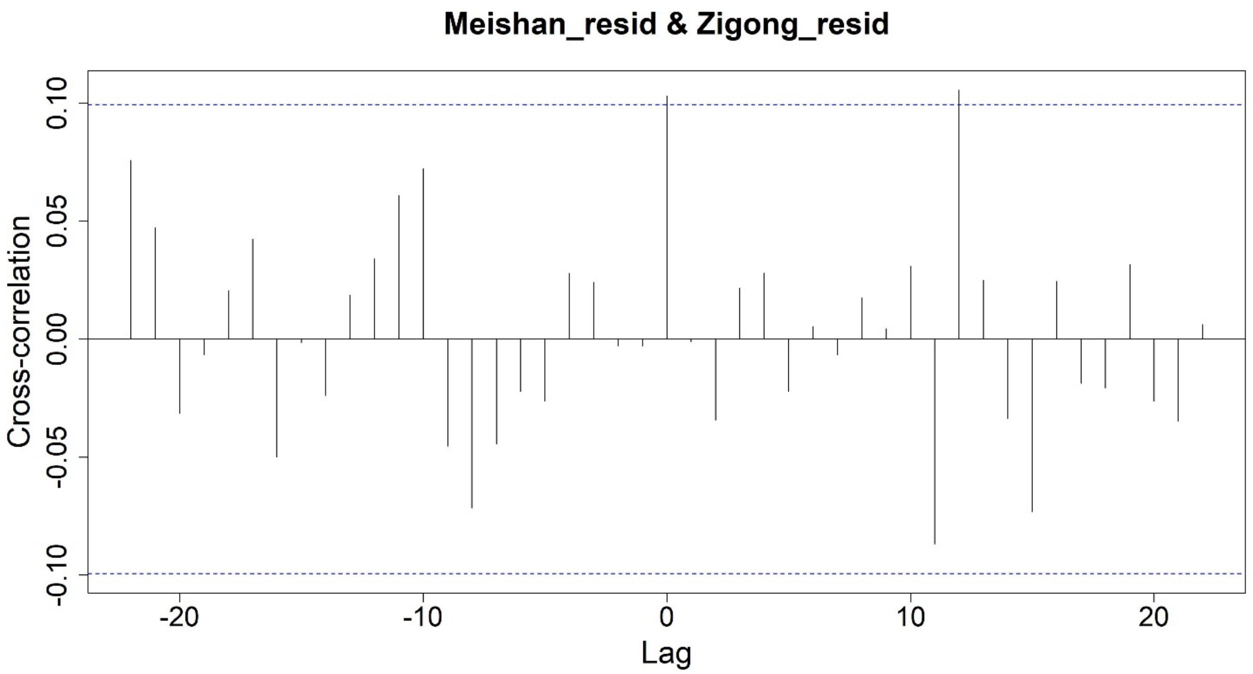

Supplementary Figure 12. The cross-correlation of the residuals between cities of cluster 5.
